# Integrating weather indices with field performance of novel fungicides for management of late blight of potato (*Phytophthora infestans*) in North Eastern Himalayan Region of India

**DOI:** 10.1371/journal.pone.0310868

**Published:** 2024-12-05

**Authors:** Utpal Dey, Shatabhisa Sarkar, Mukesh Sehgal, D. P. Awasthi, Biman De, Pranab Dutta, Saikat Majumdar, Prasenjit Pal, Subhash Chander, Ph. Ranjit Sharma, A. K. Mohanty

**Affiliations:** 1 Krishi Vigyan Kendra, Sepahijala, Latiacherra, CAU(I), Tripura, India; 2 ICAR- National Research Centre for Integrated Pest Management, Mehrauli, New Delhi, India; 3 College of Agriculture, Tripura, Lembucherra, West Tripura, India; 4 College of Post-Graduate Studies in Agricultural Sciences, CAU(I), Umiam, Meghalaya, India; 5 Department of Rural Development, University of Science & Technology, Ri-Bhoi, Meghalaya, India; 6 College of Fisheries, Lembucherra, CAU(I), West Tripura, India; 7 Directorate of Extension Education, Central Agricultural University, Imphal, Manipur, India; 8 ICAR - Agricultural Technology Application Research Institute, Zone VII, Umiam, Meghalaya, India; ICAR - Indian Agricultural Research Institute, INDIA

## Abstract

The hemibiotrophic fungus-like oomycete phytopathogen, *Phytophthora infestans* (Mont.) de Bary, causing late blight disease of potato, is one of the most serious foliar diseases of potato. The pathogen spread very rapidly and can infect at any stage of crop growth.The field experiments were carried out during winter (*rabi*) season of 2020–21 and winter (*rabi*) season of 2021–22 to find out the correlation between the disease progress and environmental factors and the effective novel fungicides registered under Central Insecticide Board and Registration Committee (CIB&RC) against *P*. *infestans*. Results revealed that T7: Mandipropamid 23.4% SC @ 0.1% (1.0 ml/L) at 35 & 55 days after sowing (DAS) and Ametoctradin 27% + Dimethomorph 20.27% SC @ 0.1% (1.0 ml/L) at 45 & 65 DAS recorded least average per cent late blight disease incidence (PLBDI) of 13.00 and 9.33, per cent late blight disease severity/index (PLBDS) of 8.81 and 5.96 and maximum tuber yield of 21.58 and 21.86 t/ha with highest benefit cost ratio (BCR) value of 1:1.95 and 1: 1.99 as compared to control during winter (*rabi*) season of 2020–21 and winter (*rabi*) season of 2021–22, respectively. T7 exhibited minimum Area under the Disease Progress Curve (AUDPC) value during both the consecutive seasons. The disease is positively correlated with maximum and minimum temperature, morning and evening relative humidity and sunshine hours. Linearity assumption scatter matrix indicates coefficient of determination of 0.916 was calculated using the pooled data.The relative potato tuber yield loss ranged from 7.38 to 19.96% and 7.14 to 19.62% during 2020–21 and 2021–22, respectively. Spray schedule with contact fungicide followed by systemic/translaminar + contact fungicide recorded reduced potato late blight disease with highest BCR value under natural epiphytotic condition.

## 1. Introduction

Potato (*Solanum tuberosum* L.) belongs to family Solanaceae, is an economically important vegetable crop grown in North Eastern Region (NER) of India [1]. NER comprises of eight states *viz*., Mizoram, Sikkim, Assam, Manipur, Arunachal Pradesh, Nagaland, Meghalaya and Tripura. The crop is also known as ‘The king of vegetables’ due to its versatile use [2]. It is a food-security crop [3] and a good source of vitamins and minerals [4]. The crop has emerged as world’s fourth-largely non-cereal food crop after paddy, wheat and corn [5]. In India, the cultivated area under potato is 2.16 million hectares (ha) with 51.3 million (m) tons (t) production and 23.77 t/ha productivity [6]. In NER, among all the states, Assam leads in area while the state of Tripura leads in productivity of potato. Tripura state produced 132764 m t from 7331 ha area [7]. Nevertheless, to increase economic growth and agricultural production in this region, the crop is one of the potential crops [8,9].

Biotic stresses are one of the important constraints to potato cultivation. Among the biotic stresses, late blight of potato poses a significant threat to production [10]. The fungus-like oomycete, *Phytophthora infestans* (Mont.) de Bary, causing late blight disease is one of the most serious and destructive disease of potato crop [11], which affects solanaceae family crops *viz*., potato (*Solanum tuberosum*), tomato (*Solanum lycopersicum*) etc. [12,13] and cause qualitative and quantitative losses [14]. Notably, the pathogen is one of the most aggressive plant pathogen [15] with a wide host range [16]. In India, the disease cause yield losses upto 100% [17,18], whereas, in Meghalaya 100% blight severity with 13.56 t/ha yield was reported in susceptible cultivar Kufri Jyoti [19]. The pathogen can destroy the whole crop within 10–15 days under favourable environmental conditions (moderate temperature and high relative humidity) [20].

Management of late blight of potato through antagonistic microorganisms or biological control agents (BCAs) is one of the alternative options [21–25]. Nonetheless, the application of BCAs is potential candidate for controlling of crop pathogens and also environmentally safe but BCAs alone not sufficient enough to provide complete management under field conditions [26]. Under variable environmental factors *viz*., relative humidity, fluctuation in day night temperature, duration of rainfall etc, the BCAs may show less effective against phytopathogens [27,28]. BCAs have slow antagonistic effect, poor performance in non-native environment [29] and less effective as compared to agrochemicals [30]. Management of potato late blight using different fungicides *viz*., mancozeb 75% WP, carbendazim 12% + mancozeb 63% WP, metalaxyl 8% + mancozeb 64% WP, cymoxanil 8%+ mancozeb 64% WP, hexaconazole 4% + zineb 68% WP, dimethomorph 50% WP, mefenoxam 8% + mancozeb 64% WP, chlorothalonil 75% WP, iprovalicarb 5.5% + propineb 61.25% WP, thiram 75% DS, copper oxychloride 50% WP, carboxin 37.5% + thiram 37.5% WS, metalaxyl 35% WS, chlorothalonil 33% + metalaxyl 3.3% SC, zoxystrobin 23% SC, thiophanate methyl 70% WP, ethaboxam 40% SC, mancozeb 60% + dimethomorph 9% WP, metiram 55% + pyraclostrobin 5% WG, famoxadone 16.6% + cymoxanil 22.1% SC, were reported from different parts of India [31–35]. Furthermore, these fungicides were used alone and in combination for the management of the disease. Notably, widely used fungicide, phenylamide compound metalaxyl [36], mefenoxam [37] were used earlier for the management of potato late blight but now the pathogen has build up resistant against the fungicide. Hence, it is necessary to find out a broad spectrum systemic and contact fungicide with protective and curative action to manage the disease.

Owing to the importance of the polycyclic disease, potential yield losses and understanding the linkages among weather indices, and disease progress, the present investigation was undertaken to find out the correlation and the effective novel fungicides registered under CIB&RC against *P*. *infestans*.

## 2. Materials and methods

### 2.1 Study site

The experiment was carried out during 2020–21 and 2021–22 at Pathalia GP, Bishalgarh block, Sepahijala district, Tripura, India. The experimental area was located approximately 8.9 km from Bishalgarh and 31 km from Agartala (capital of Tripura state, 23.694058° latitude, 91.336128° longitude).The soil texture of the experiment site was sandy clay loam and soil reaction (pH) of soil is slightly acidic in nature (6.52).

### 2.2 Weather

The weather of study area is humid sub-tropical under agro climatic zones of Humid Eastern Himalayan Region characterized by high rainfall. The humidity ranges from 42 to 100 per cent and annual rainfall ranges from 2,000 to 3,000 mm.

### 2.3 Seed materials

The potato tubers cultivar Kufri Jyoti (Susceptible) about 2.5 cm in diameter and about 30–40 gm in weight was selected for the experiment. The tubers were disease free and healthy. The potato tubers kept on sunlight sterilized sand spread over the floor covering with gunny bag for good sprouting.

### 2.4 Fungicides

A total of six fungicides *viz*., Mancozeb 75% WP, Dimethomorph 50% WP, Ametoctradin 27% + Dimethomorph 20.27% SC, Kresoxim methyl 44.3% SC, Mandipropamid 23.4% SC, Metiram 55% + Pyraclostrobin 5% WG were used in both the experiment.

### 2.5 Meteorological parameters

**Fig 1:**
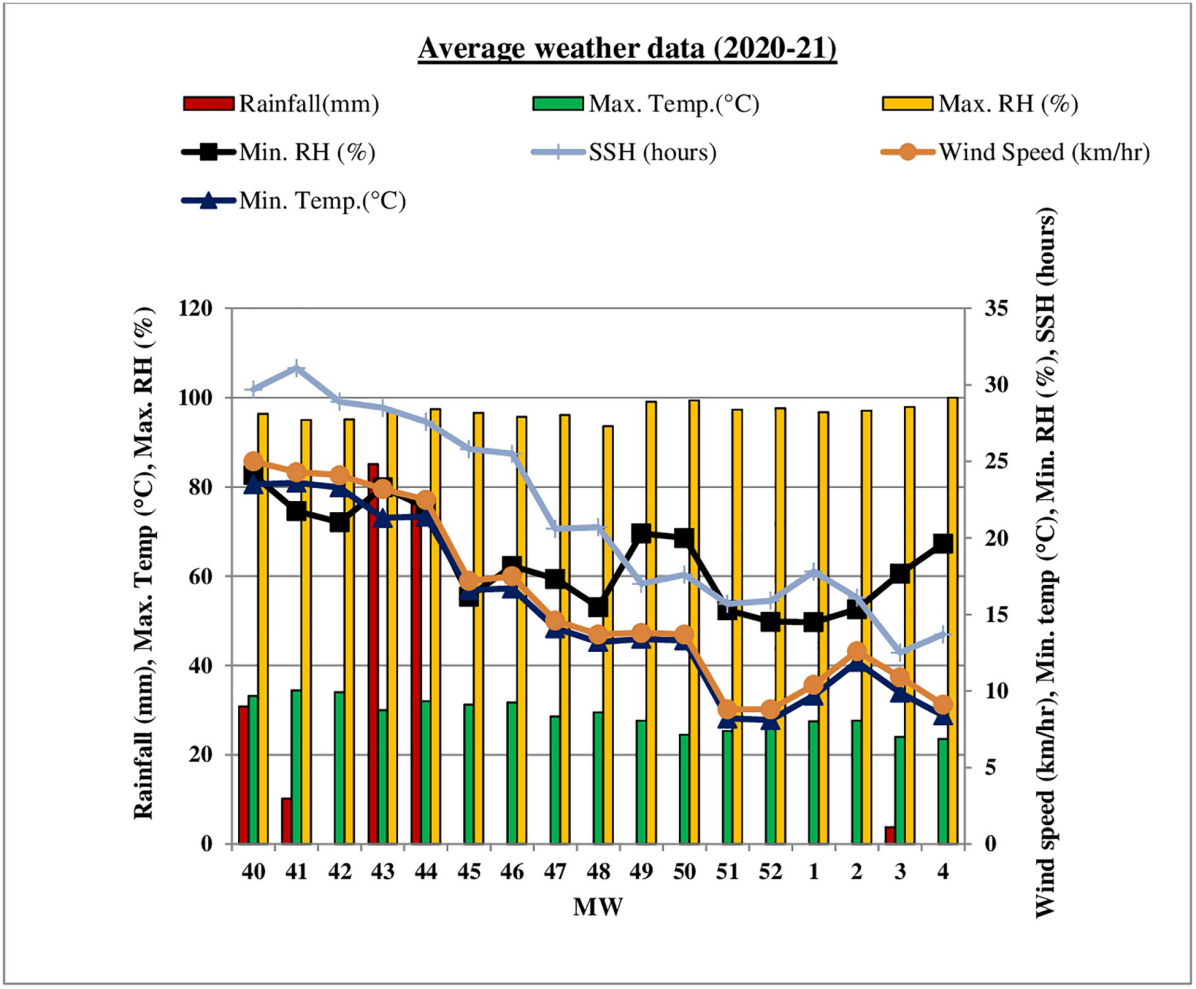
Meteorological parameters during winter (*rabi*) season of 2020–21.

**Fig 2:**
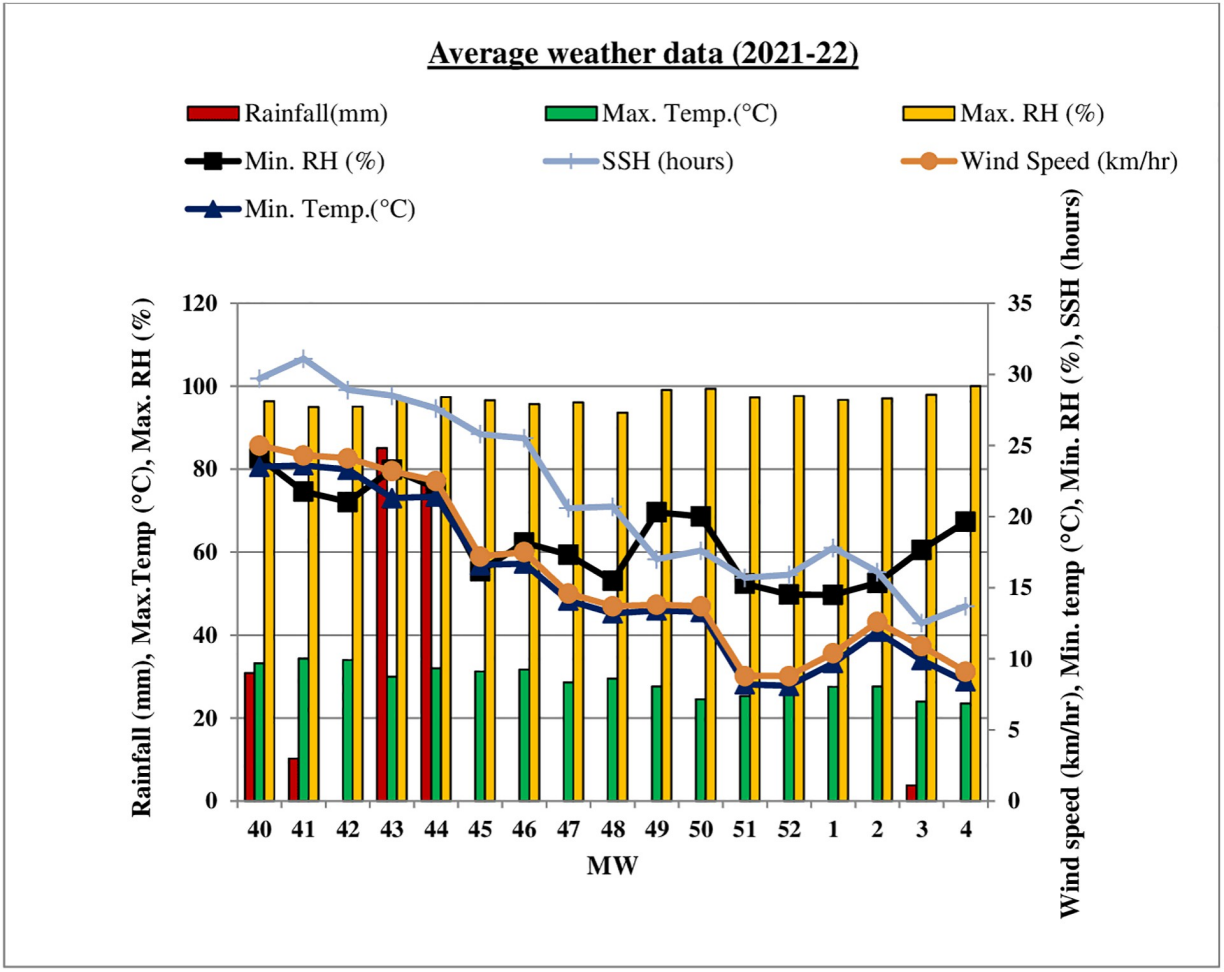
Meteorological parameters during winter (*rabi*) season of 2021–22.

Meteorological parameters (temperature, relative humidity, rainfall, wind speed, sunshine hours) for the experiment were obtained from Indian Council of Agricultural Research (ICAR) Research Complex for NEH Region, Tripura Centre, Lembucherra, West Tripura and correlation was worked out (Figs 1 & 2).

### 2.6 Experiment details

A field experiment was carried out under ICAR- National Research Centre for Integrated Pest Management, New Delhi sponsored project during winter (*rabi*) season of 2020–21 in experiment 1 and winter (*rabi*) season of 2021–22 in experiment 2. Plot size of 3.4×3 sq. m at spacing of 50×15 sq. cm was taken for both the experiment. Tubers were planted on top of the ridges in the first week of October. Nitrogen (N), phosphorous (P) and potassium (K) fertilizers were applied @ 150:100:100 kg ha^-1^. Lime was applied @ 500 kg ha^-1^. Half dose of N along with full doses P and K were applied at time of sowing as basal dose and rest half dose of N applied at 30 days after sowing (DAS) through top dressing. Two times earthing up operation followed (25 and 40 DAS). Light irrigation was given at 6 DAS and second irrigation at 30 DAS (20.4 and 12.8 cm rainfall received during October, 2020–21 and 2021–22, respectively).The subsequent furrow irrigations were given up to 5 cm depthat an interval of 15 days for 3 times. The experiment was laid out following a randomized block design with 3 replications and 8 treatments in participatory mode in the farmer’s field. An equal amount of water sprayed in control plot. At the time of fungicide application, plastic sheet was used to reduce drift problem. The treatment details are provided in Table 1.

**Table 1:** Different treatment details and doses.

Treatment
T1: Metiram 55% + Pyraclostrobin 5% WG @ 0.1% (1.0 g/L) at 35, 45 (tuberization initiation stage), 55 & 65 (tuber filling stage) DAS
T2: Mancozeb 75% WP @ 0.25% (2.5 g/L) at 35 & 55 DAS and Dimethomorph 50% WP @ 0.1% (1.0 g/L) at 45 & 65 DAS
T3: Mandipropamid 23.4% SC @ 0.1% (1.0 ml/L) at 35 & 55 DAS and Dimethomorph 50% WP @ 0.1% (1.0 g/L) at 45 & 65 DAS
T4: Mancozeb 75% WP @ 0.25% (2.5 g/L) at 35 & 55 DAS and Kresoxim methyl 44.3% SC @ 0.1% (1.0 ml/L) at 45 & 65 DAS
T5: Mandipropamid 23.4% SC @ 0.1% (1.0 ml/L) at 35 & 55 DAS and Kresoxim methyl 44.3% SC @ 0.1% (1.0 ml/L) at 45 & 65 DAS
T6: Mancozeb 75% WP @ 0.25% (2.5 g/L) at 35 & 55 DAS and Ametoctradin 27% + Dimethomorph 20.27% SC @ 0.1% (1.0 ml/L) at 45 & 65 DAS
T7: Mandipropamid 23.4% SC @ 0.1% (1.0 ml/L) at 35 & 55 DAS and Ametoctradin 27% + Dimethomorph 20.27% SC @ 0.1% (1.0 ml/L) at 45 & 65 DAS
T8: Control

### 2.7 Disease evaluation

#### 2.7.1 PLBDI

PLBDI was recorded by counting the infected plants showing late blight disease symptoms and PLBDI was recorded at 50, 57, 64 and 71 days after sowing (DAS) at 7-day intervals from the center of three middle rows of each plot. PLBDI was calculated by using formula [38].


$$PLBDI=\frac{Number\;of\;plant\;infected\;}{Total\;number\;of\;plant\;examined\;}\times 100$$

#### 2.7.2 PLBDS

Based on numerical rating/scores observed, PLBDS was calculated by applying the formula [39] as given below.


$$PLBDS=\frac{Summation\;of\;numerical\;ratings}{No.of\;leaves/plants\;observed\;\times Maximum\;rating}\times 100$$

In each plot 50 plants were selected randomly and in each plant one top leaf, one middle leaf and one lower leaf were selected from the center of three middle rows of each plot for estimation of severity of the disease. The data on severity of the disease was recorded before every spray by using 0–9 rating scale (Table 2) [40].

**Table 2:** The 0–9 rating scale for the assessment of late blight of potato leaves.

Severity scale	Rating grade (%)	Level of resistance/ susceptibility
0	0	No disease lesion
1	10	A small lesion on the leaves less than 10% area coverage of the whole leaflet
3	11–20	Lesion area between 10–20% of the whole leaflet
5	21–30	Lesion area between 20–30% of the whole leaflet
7	31–60	Lesion area between 30–60% of the whole leaflet
9	Over 60	Lesion area over 60% of the whole leaflet

#### 2.7.3 Per cent disease reduction over control (PDC)

Further, PDC was worked out by applying the formula:

$$PDC=\frac{PDI\;in\;control\;plot-PDI\;in\;treatment\;plot}{PDI\;in\;control\;plot}\times 100$$

#### 2.7.4 AUDPC

AUDPC values were calculated applying the formula [41] as given below.

$$\text{AUDPC}=\sum_{i=0}^{n-1}0.5(x_{i+1}+x_{i})(t_{i+1}-t_{i})$$

where Xi is the cumulative disease severity expressed as a proportion at the i^th^ observation, ti is the time (days after planting) at the i^th^ observation, and n is total number of observations. AUDPC values were expressed in percent-days.

#### 2.7.5 Coefficient of disease index (CODEX)

CODEX value was also calculated by the formula [42]

$$CODEX=\frac{Per\;cent\;disease\;incidence\;\times \;Per\;cent\;disease\;index\;}{100}$$

### 2.8 Yield analysis

The crop was harvested at its physiological maturity and treatment-wise tuber yield was recorded from central rows of each plot. The yield was converted into hectare basis.

### 2.9 Yield loss estimation

The relative potato tuber yield loss (RPTYL) due to late blight disease was calculated by the formula [43]

$$RPTYL=\frac{YP-YT}{YP}\times 100$$

Where, YP is yield from protected plot, YT is yield from unprotected plot:

### 2.10 Economic analysis

Economic analysis *viz*., gross cost of cultivation (Rs/ha), gross return (Rs.), additional income/ha over control (Rs.), net return (Rs.) and BCR was calculated. BCR is calculated by dividing total net income by gross cost of cultivation.

### 2.11 Data analysis

PLBDI, PLBDS, PDC, AUDPC, CODEX and yield data was recorded separately. However, treatment-wise yield data (tuber yield) was recorded at its physiological maturity stage. The PLBDI and PDI data were arc-sine transformed before analysis. The data collected were subjected to statistical analysis linearity assumption scatter matrix with normal distribution analyzed by using SPSS (Version 1.6). Each treatment Standard error of means [S.Em (±)] and Least Significant difference (LSD) at 0.05 probabilities (p = 0.05) were calculated [44].

## 3. Results

**Fig 3:**
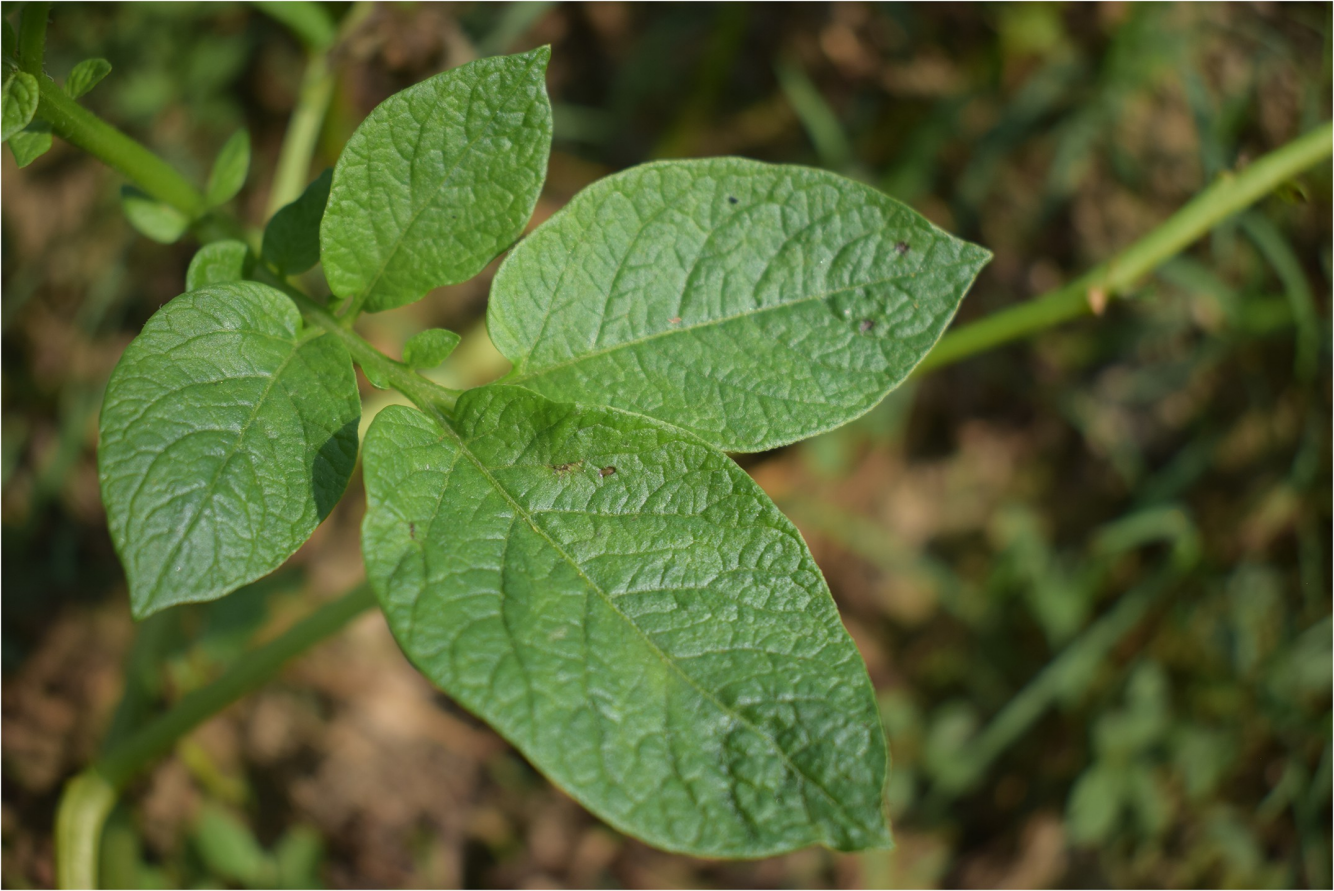


**Fig 4:**
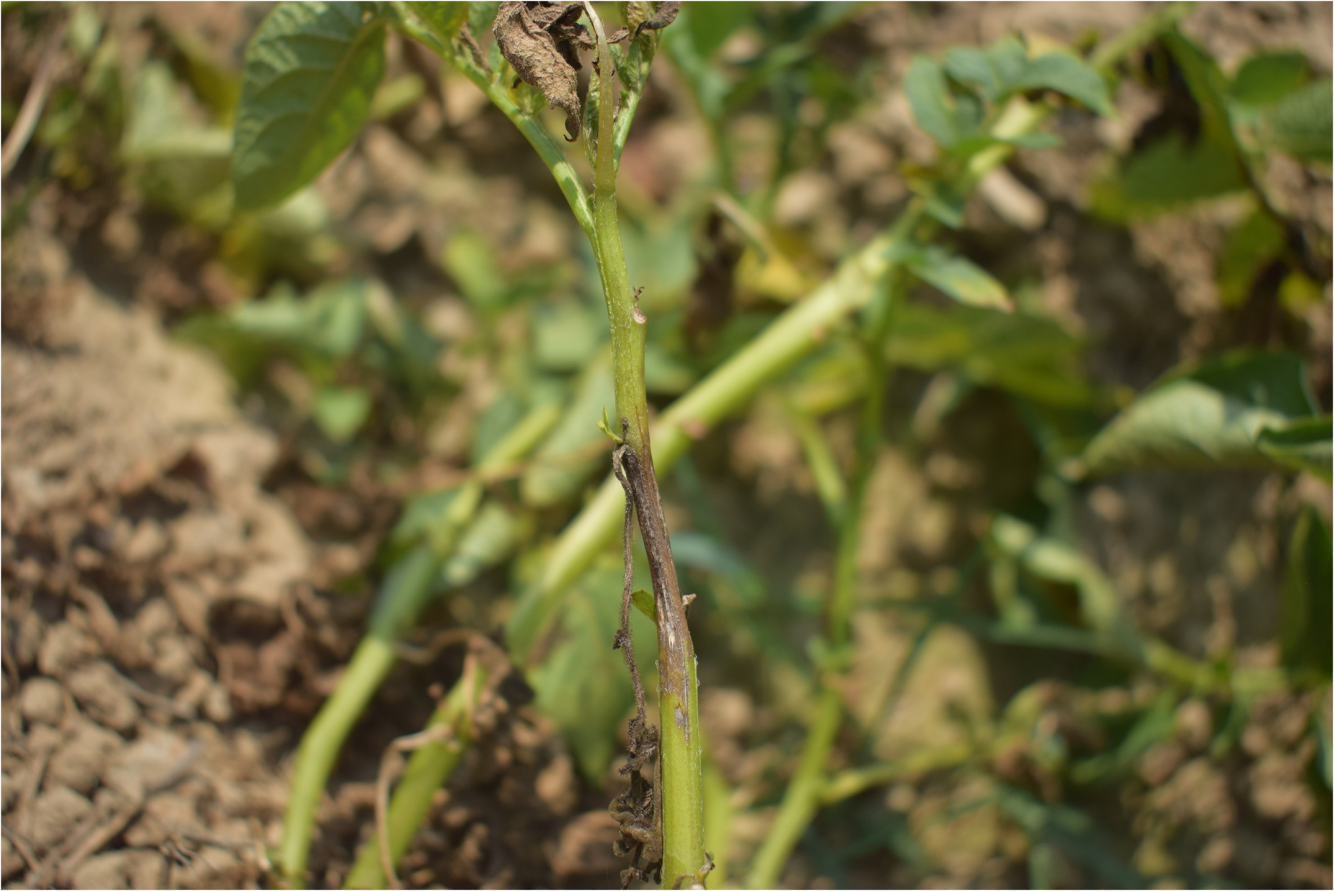


**Fig 5:**
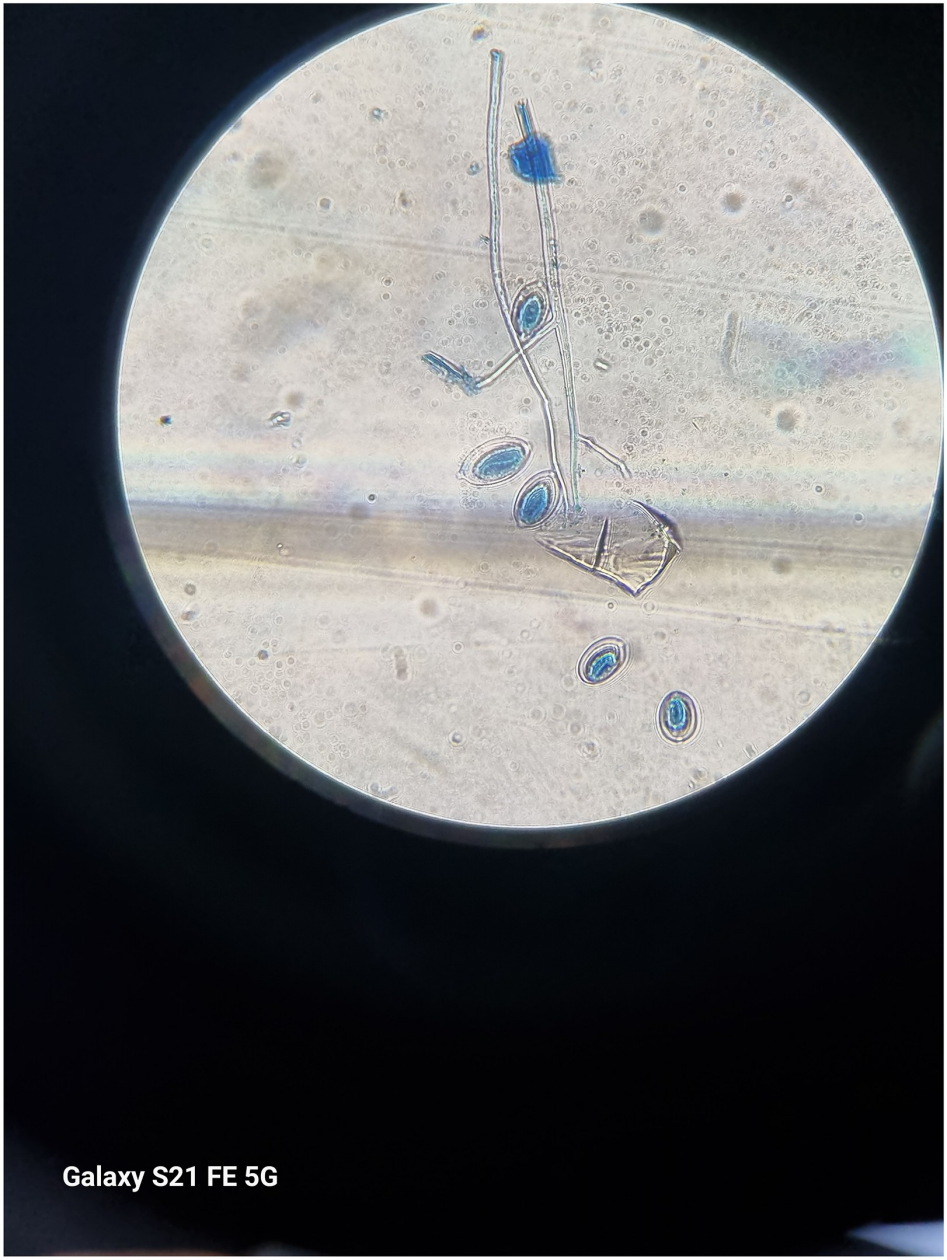


**Fig 6:**
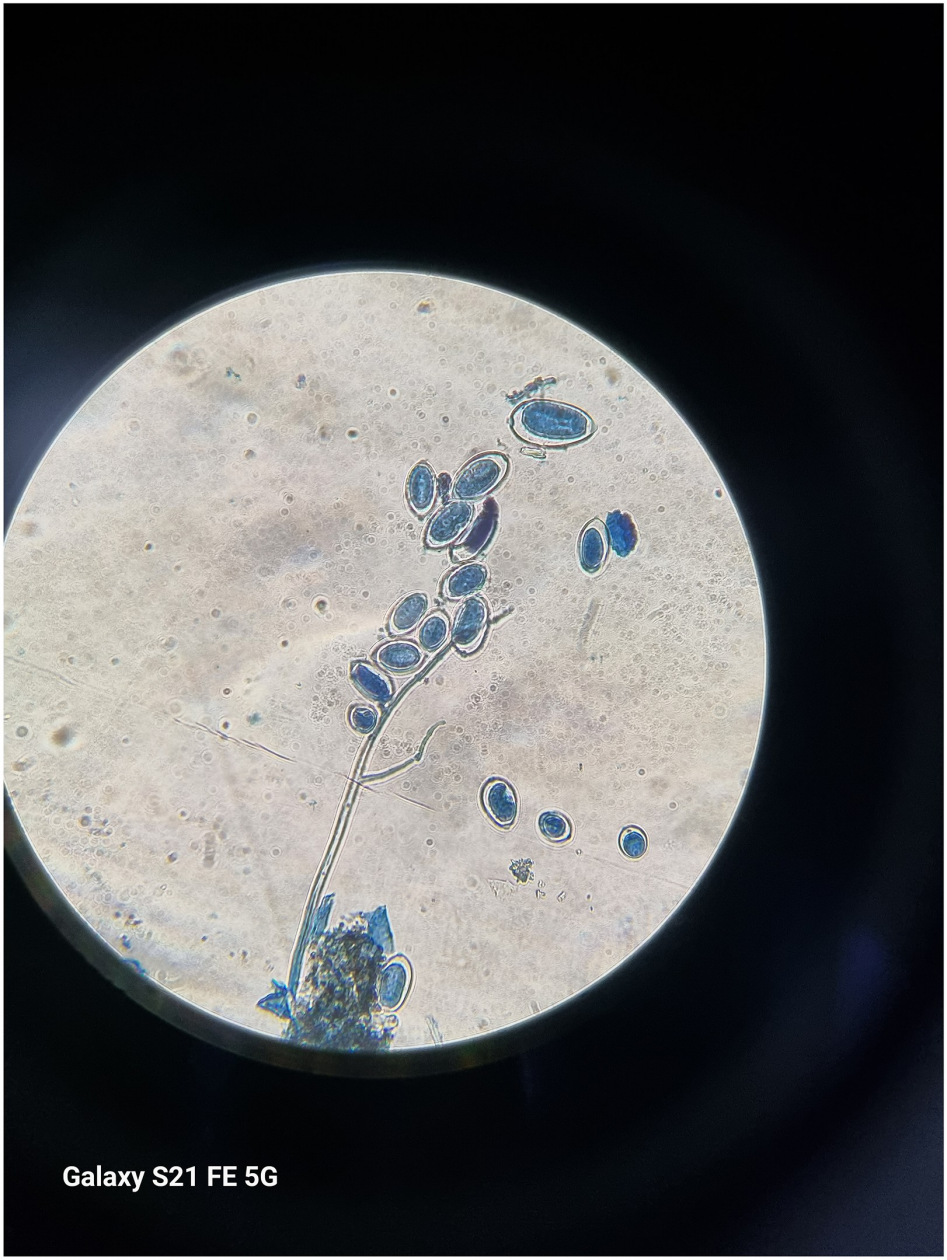


Under field conditions, late blight disease symptoms appeared as small dark green water-soaked spot on lower leaves of the potato plants (Fig 3). The affected spots start from edges or tips of leaves and vary from circular to irregular-shaped. The necrotic spots spread throughout the leaf area under favorable conditions. In advance stages, the disease affect the stems (Fig 4) and the whole plant dry out. From the whitish cottony growth found on abaxial surface of leaf, lemon shaped sporangium (Fig 5) and sporangiophore observed under microscope (Fig 6).

### 3.1 Effect of weather parameters on PLBDI and PLBDS

**Fig 7:**
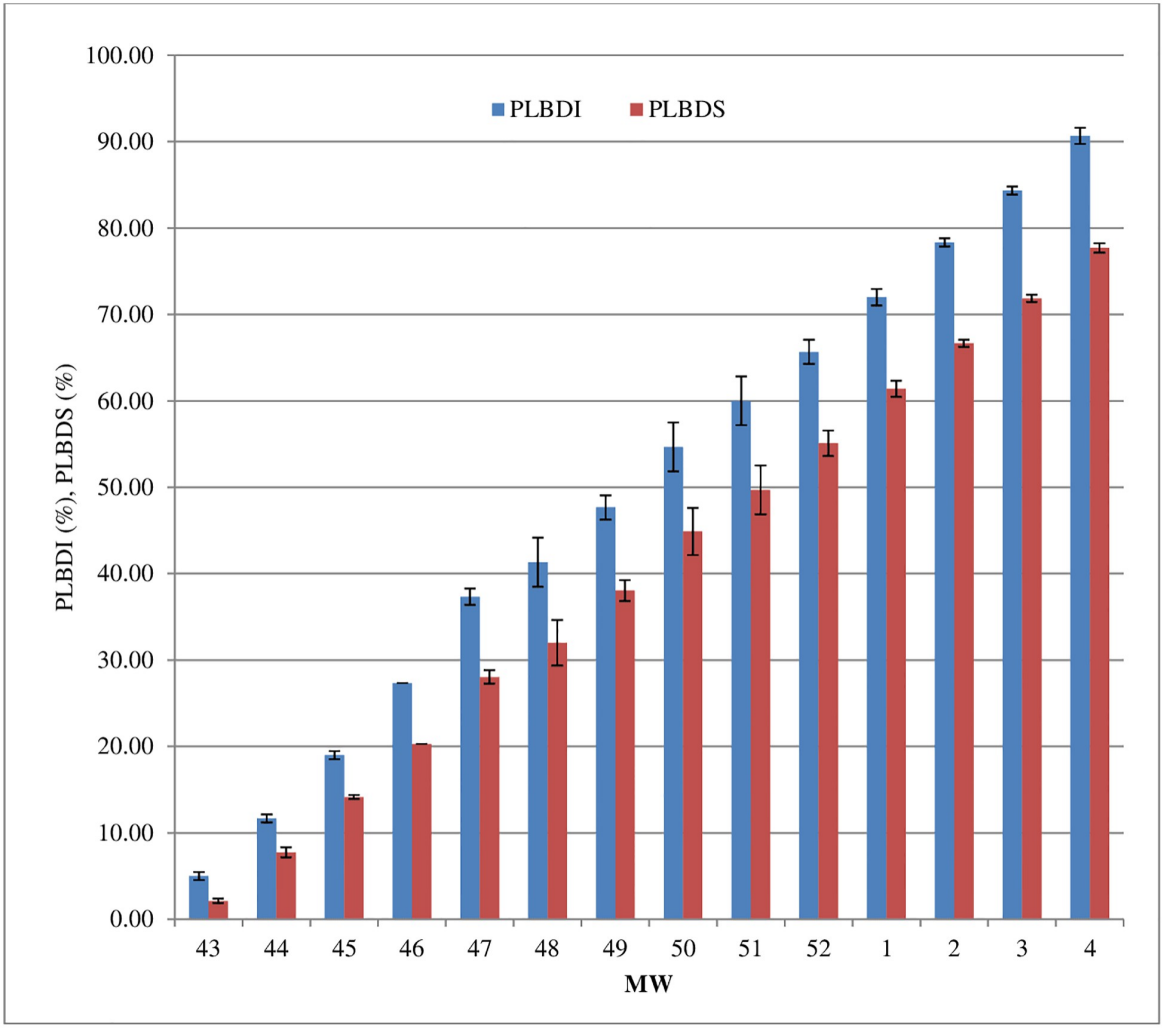
Effect of weather parameters on PLBDS (Pooled data of 2020–21 and 2021–22). *Error bar represent standard error of the mean.

Results infers that different weather variables *viz*., temperature (maximum & minimum), relative humidity (morning & evening), wind speed and sunshine hours play an crucial role in the development of the disease. The average PLBDI and PLBDS in the range of and 5.0 to 90.67% and 2.12 to77.70% were recorded at 43 to 4 MW during winter (*rabi*) season of 2020–21 (Fig 7).

At the time of the disease initiation, the average minimum and maximum temperature were recorded as 20.3°C and 30.7°C, respectively, with morning and evening relative humidity levels of 96.7% and 72.9%. During this period, 42.6mm rainfall, 0.9 km h^-1^ wind speed and 5.6 hours sunshine hours was also observed. The disease is positively correlated with maximum and minimum temperature, evening relative humidity, morning relative humidity, and sunshine hours.

**Fig 8:**
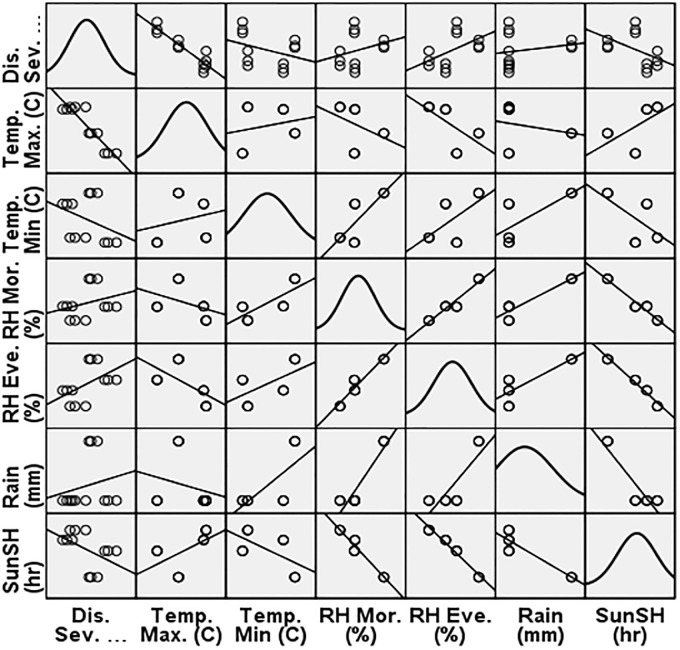
Linearity assumption scatter matric with normal distribution diagonally of control plot.

The model summary of the control plot reveals that the coefficient of determination, derived using the pooled data, is 0.916. The regression analysis indicates that 91.6% (R^2^ = 0.916) of the variation in disease severity can be explained by the duration of temperature (highest and lowest) and relative humidity (morning) (Fig 8). Given that the p-value is below 0.05, it may be deduced that the regression model is statistically significant. However, the variables of relative humidity (evening), rainfall, and sunlight hours were excluded from the model due to their strong relationships with other predictor variable(s). The regression model demonstrated a positive correlation between the severity of disease and meteorological markers, namely temperature and relative humidity. The coefficient of determination (R value) for the regression model assessing linearity assumption is 0.957.

Thus, the multicollinearity of relative humidity (evening), rainfall and sunshine hours reduces the precision of the estimated coefficients, which weakens the statistical power of regression model.

The model accurately predicts the dependent variable, which is the severity of the disease is statistically significant with temperature (maximum and minimum) and relative humidity (morning) but differed insignificantly with relative humidity (evening), rainfall and sunshine hours.

**Fig 9:**
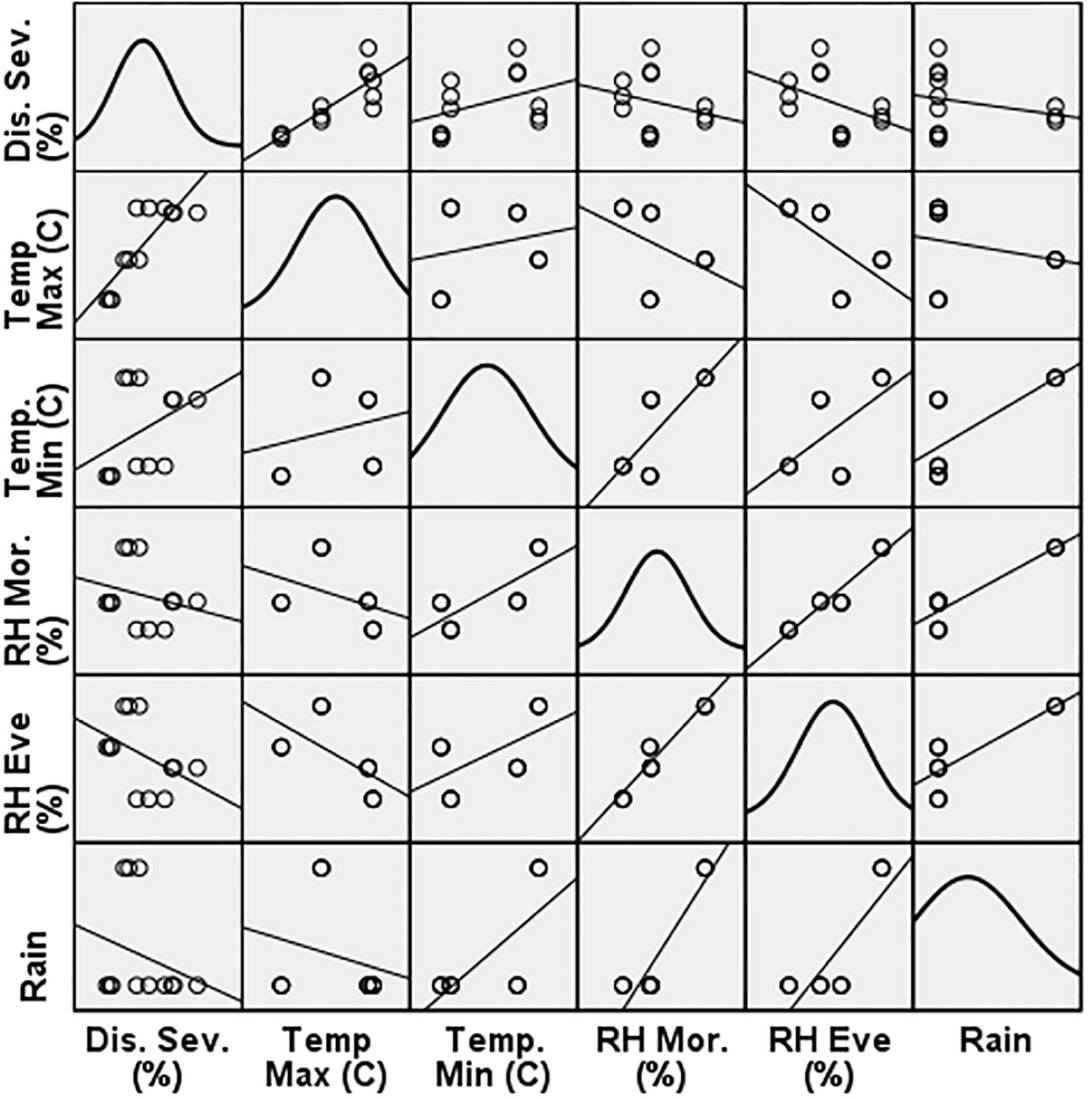
Linearity assumption scatter matrix with normal distribution diagonally of treatment plot (T_7_).

The model summary of T7 treatment shows the coefficient of determination of 0.899 was calculated using the pooled data. The regression study shows that 89.9% (R^2^ = 0.899) of the variability in disease severity can be attributed to the duration of temperature (maximum and minimum) and relative humidity (morning) (Fig 9). Since the p-value is less than 0.05, there is sufficient evidence to conclude that the regression model is statistically significant. Nonetheless, relative humidity (evening), rainfall and sunshine hours were dropped from the model because of high correlations with other predictor variable(s). The regression R value of the linearity assumption is 0. 948.

Hence, the presence of multicollinearity among the variables of relative humidity (evening), rainfall, and sunshine hours diminishes the accuracy of the estimated coefficients, thereby compromising the statistical strength of the regression model. Consequently, relying on the p-values to identify independent variables that are statistically significant may not be reliable.

### 3.2 AUDPC

**Fig 10:**
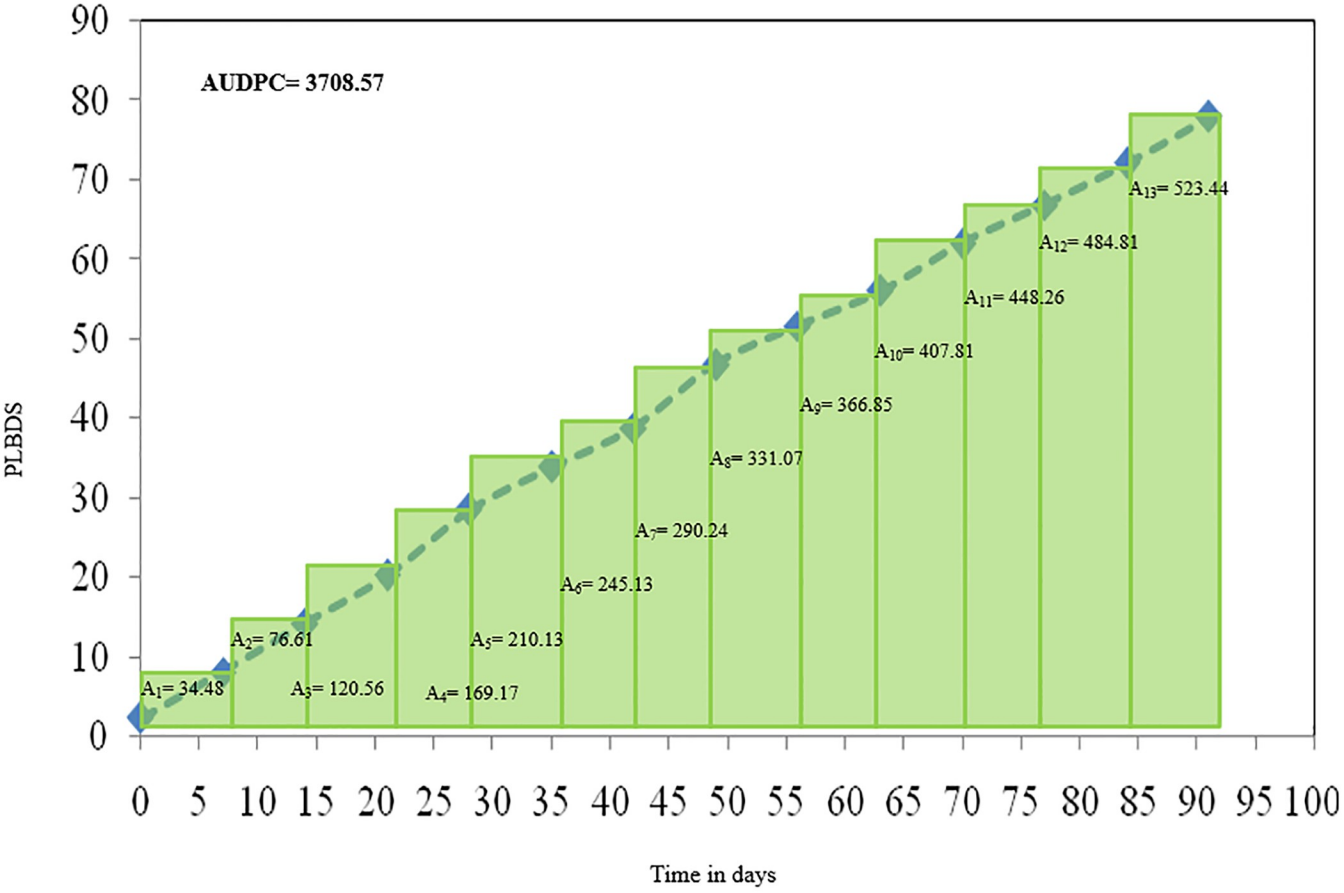
Effect of weather parameters on AUDPC (Pooled data of 2020–21 and 2021–22).

Pooled data presented in Fig 10 revealed that highest AUDPC value (523.44) was recorded at 4^th^ MW. AUDPC value is correlated with disease severity. Total AUDPC value of 3780.78 and 3636.37 were observed in two consecutive seasons. The higher AUDPC value denotes the higher disease pressure over time. Results exhibited that AUDPC value (A-value) increases with an increase in the age of the crop. The disease spreads rapidly from infected plant to healthy plant. The more necrotic foliage area under more AUDPC value was noticed both the consecutive seasons.

### 3.3 Effect of fungicides on PLBDI

**Fig 11:**
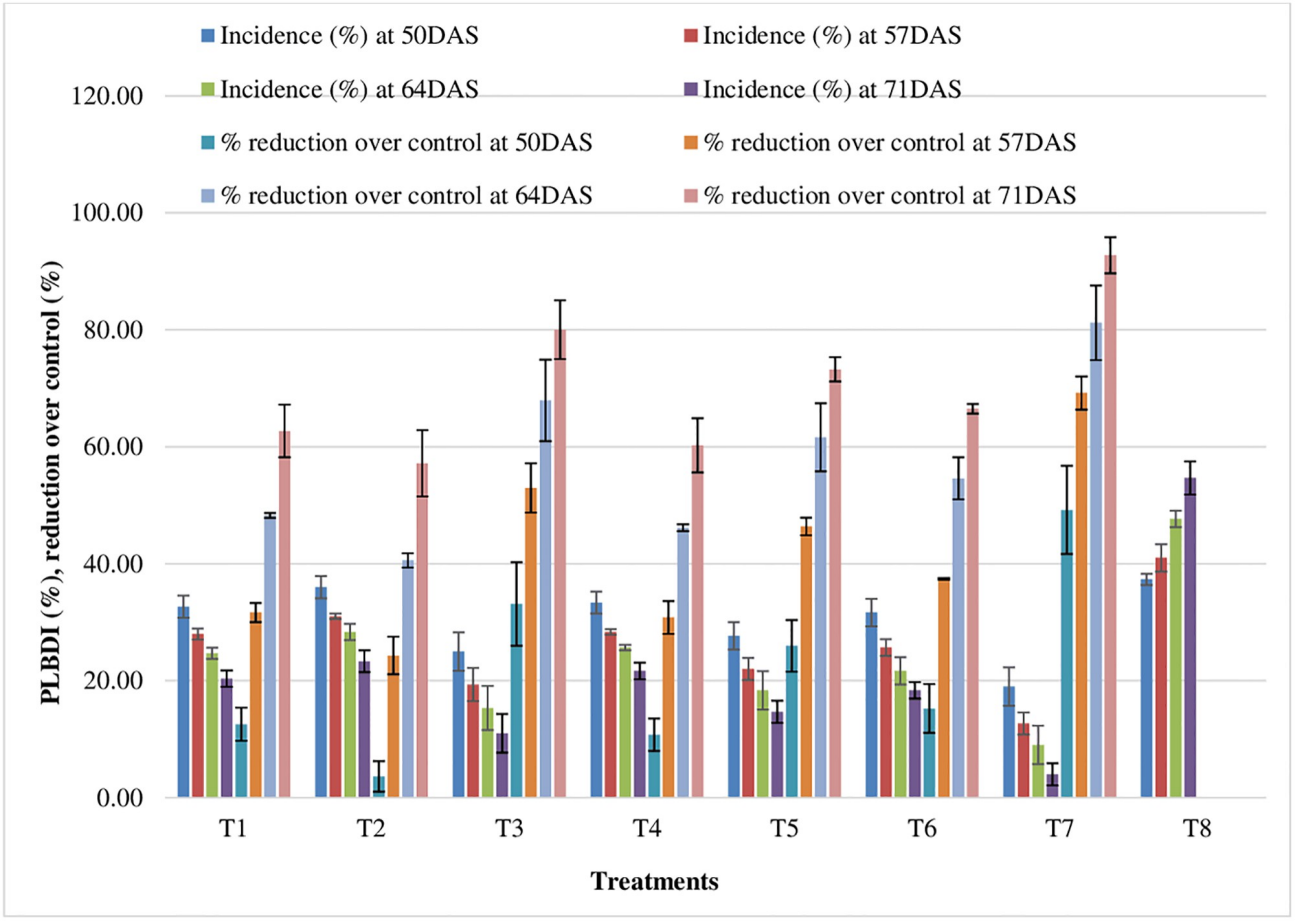
Effect of fungicides on PLBDI (Pooled data of 2020–21 and 2021–22). *Error bar represent Standrad Error.

To find out the effectiveness of different fungicides against potato late blight disease, the field evaluation was carried out during winter (*rabi*) season of 2020–21 and 2021–22. The application of different fungicides evaluated under natural epiphytotics revealed that all the fungicides were superior as compared to control and showing decreasing trends of PLBDI. Results revealed that among the treatments, T7 recorded least PLBDI of 21.33, 14.00, 11.33 and 5.33 at 50, 57, 64 and 71 DAS, respectively during winter (*rabi*) season of 2020–21. However, during winter (*rabi*) season of 2021–22, least PLBDI of 16.67, 11.33, 6.67 and 2.67 were found under T7 at 50, 57, 64 and 71 DAS, respectively. The average PLBDI of 13.00 and 9.33 were noticed during 2020–21 and 2021–22, respectively. Furthermore, pooled analysis revealed that the average PLBDI value ranged from 11.17 to 45.17 at different treatment plots (Fig 11). The second best treatment was T3 which recorded PLBDI of 27.33, 21.33, 18.00 and 13.33 during 2020–21 and 22.67, 17.33, 12.67 and 8.67 during 2021–22 at 50, 57, 64 and 71 DAS, respectively, which were statistically at par with each other. The highest average potato late blight disease reduction (pooled analysis) of 73.09% was obtained from T7 over control. The average late blight disease reduction over control ranged from 41.93 to 69.59 and 44.93 to 76.60 during 2020–21 and 2021–22, respectively.

### 3.4 Effect of fungicides on PLBDS

**Fig 12:**
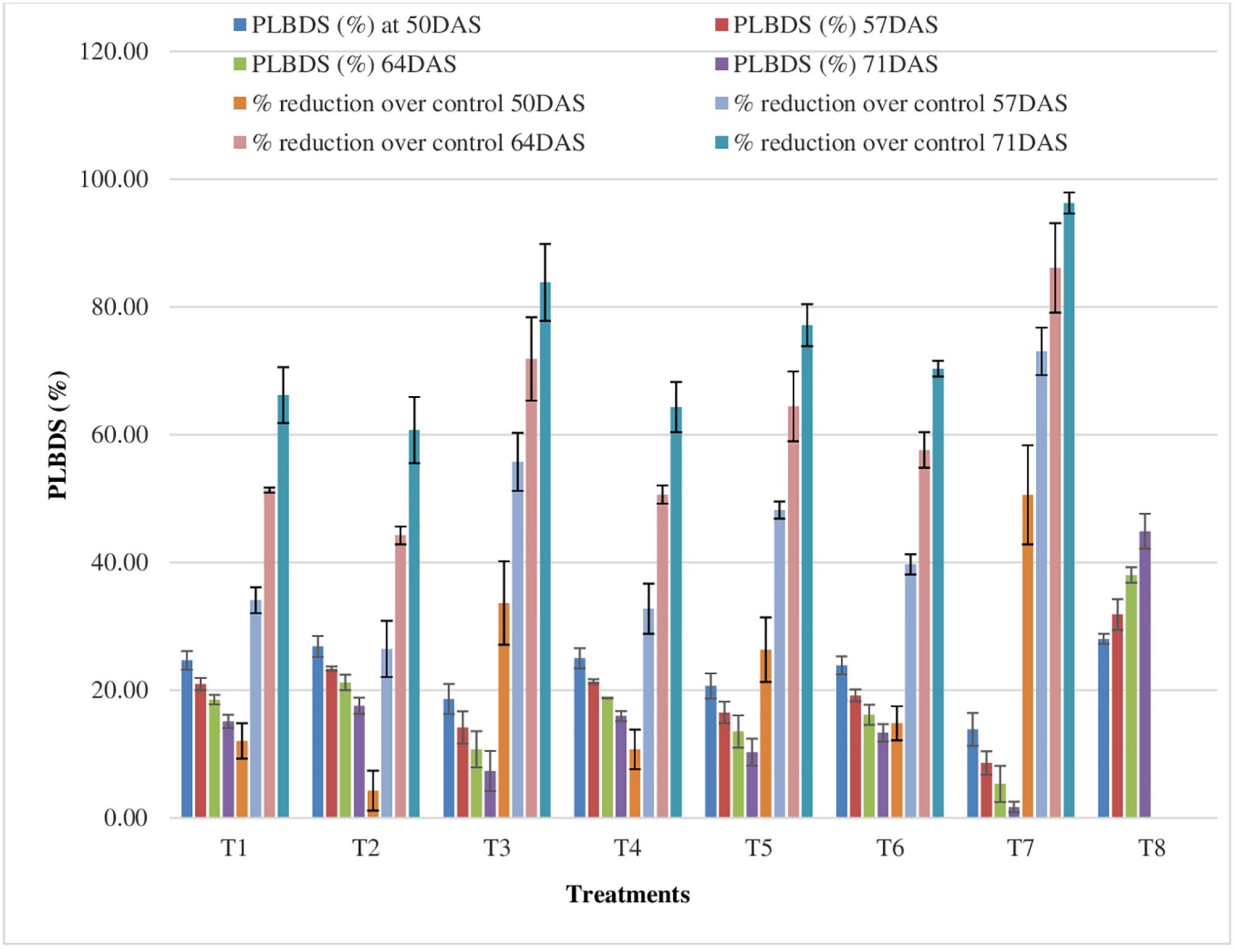
Effect of fungicides on PLBDS (Pooled data of 2020–21 and 2021–22). *Error bar represent Standrad Error.

Data (pooled) depicted in Fig 12 revealed that least PLBDS of 13.89, 8.63, 5.33, and 1.70 was recorded with T7 as compared to control. It was followed by T3 with PDI of 18.63, 14.15, 10.74 and 7.33. The average PLBDS of 7.39 was noticed as compared to control (PLBDS, 35.70). T7 recorded the highest average potato late blight disease reduction of 76.49% (Fig 12). Furthermore, at different treatment plots, the average PLBDS value (pooled) varied from 7.39 to 35.70 whereas, average potato late blight disease reduction of 33.92 to 76.49%.

### 3.5 Effect of fungicides on AUDPC

**Fig 13:**
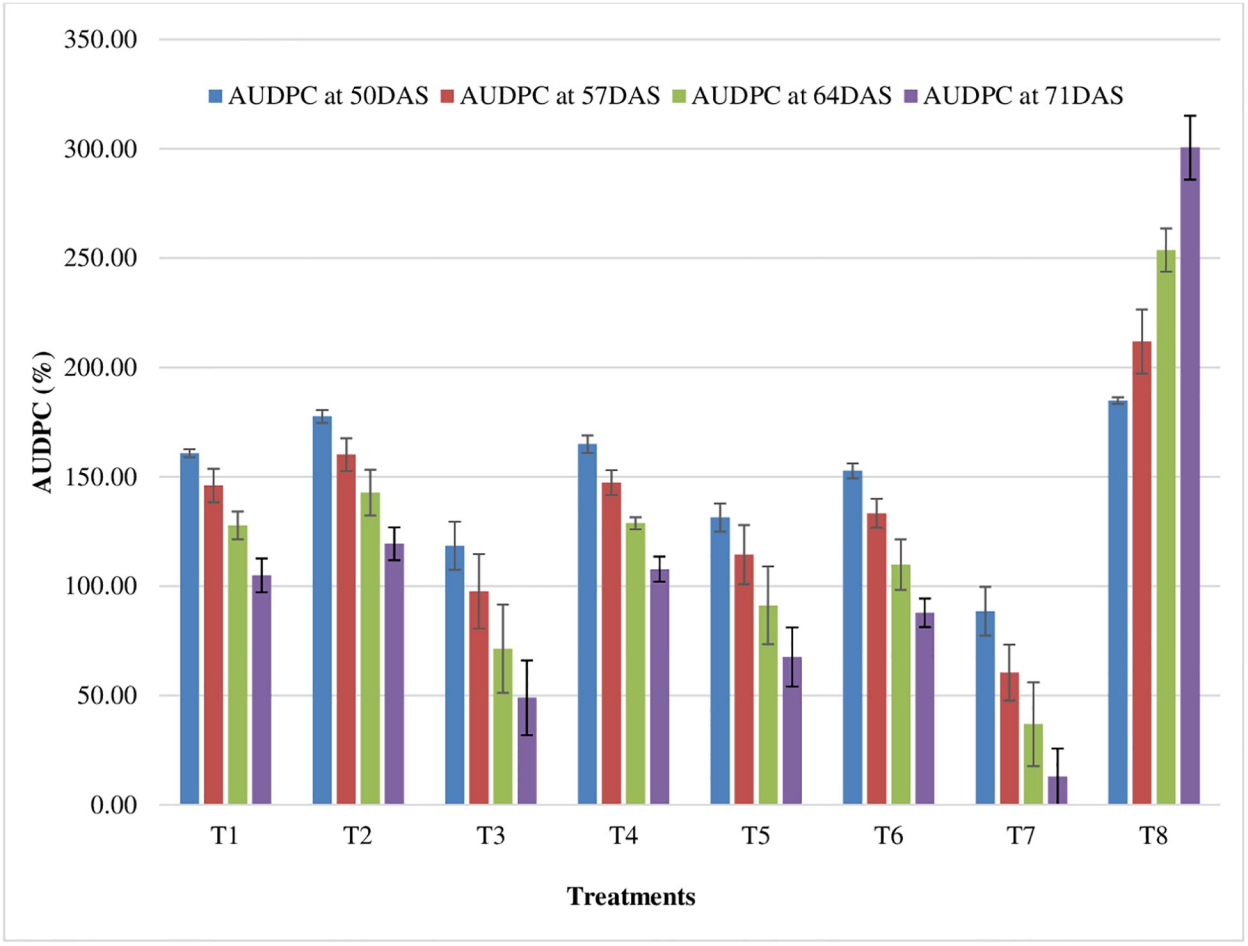
AUDPC values of fungicide variants (Pooled data of 2020–21 and 2021–22). *Error bar represent standard error of the mean.

All the fungicides applied for the experiment significantly reduced the AUDPC value at different stages of crop. Maximum AUDPC value 183.81, 222.19, 260.56 and 313.96 were recorded in control plot at 50, 57, 64 and 71 DAS, respectively during winter (*rabi*) season of 2020–21. Furthermore, during winter (*rabi*) season of 2021–22, maximum AUDPC value 185.89, 201.44, 246.56 and 287.00 were recorded in control plot at 50, 57, 64 and 71 DAS, respectively. Minimum AUDPC value of 88.54,60.41, 36.81 and 12.83 were observed with T7 in 1^st^ cropping season, whereas, AUDPC value of 184.85, 211.81, 253.56 and 300.48 in control plot at 50, 57, 64 and 71 DAS, respectively (Fig 13).

### 3.6 CODEX

Potato late blight disease was gradually reduced after the application of fungicides. Minimum CODEX value of 1.15 and 0.56 was recorded with T7 followed by T3 (CODEX value, 2.93 and 1.66) over control (CODEX value, 17.19 and 15.10) during 2020–21 and 2021–22, respectively (Table 3). The CODEX value at different treatment plots ranged from 1.15 to 17.19 and 0.56 to 15.10. The CODEX value is depends on the disease incidence and severity. Over the two years, it was evident that the CODEX value reduced after spraying of contact fungicide and translaminar/systemic + contact fungicide.

**Table 3:** Effect of fungicides sprayings on late blight incidence, severity and tuber yield in potato.

Treatments	2020–21						2021–22					
	Mean incidence	Mean severity* (%)	CODEX (%)	Tuber yield (t/ha)	% increase in yield over control	Yield loss (%)	Mean incidence	Mean severity* (%)	CODEX (%)	Tuber yield (t/ha)	% increase in yield over control	Yield loss (%)
T1	26.83 (31.20)	20.19 (26.70)	5.42	17.90	8.5	17.06	26.00 (30.66)	19.44 (26.17)	5.06	18.24	8.4	16.58
T2	30.00 (33.21)	22.59 (28.38)	6.78	17.27	4.7	19.96	29.33 (32.79)	21.91 (27.91)	6.43	17.57	4.4	19.62
T3	20.00 (26.57)	14.63 (22.49)	2.93	19.98	21.1	7.38	15.33 (23.05)	10.80 (19.18)	1.66	20.30	20.7	7.14
T4	27.50 (31.63)	20.50 (26.92)	5.64	17.60	6.7	18.43	27.00 (31.31)	20.07 (26.62)	5.42	17.90	6.4	18.13
T5	19.33 (28.20)	16.72 (24.14)	3.73	19.03	15.4	11.79	19.00 (25.84)	13.80 (21.80)	2.62	19.29	14.6	11.77
T6	25.67 (30.44)	19.07 (25.90)	4.90	18.57	12.5	13.95	23.00 (28.66)	17.20 (24.51)	3.96	18.85	12.0	13.77
T7	13.00 (21.13)	8.81 (17.27)	1.15	21.58	30.8	--	9.33 (17.79)	5.96 (14.13)	0.56	21.86	29.9	--
T8	46.50 (42.99)	36.96 (37.44)	17.19	16.50	**--**	23.54	43.83 (41.46)	34.44 (35.94)	15.10	16.82	**--**	23.04
S.Em±	**--**	**--**	**--**	**0.44**	**--**	**--**	**--**	**--**	**--**	**0.43**	**--**	--
l.s.d (*p* = 0.05)	**--**	**--**	**--**	**1.33**	**--**	**--**	**--**	**--**	**--**	**1.29**	**--**	**--**

*-means of replications, Figures in parenthesis is angular transformed value.

### 3.7 Tuber yield

The potato tuber yield was correlated with PLBDI and PLBDS. Throughout the two consecutive years, the maximum tuber yield of 21.58 and 21.86 t/ha was recorded with T7 followed by T3 (yield, 19.98 and 20.30 t/ha) over control (yield, 16.50 and 16.82 t/ha) which were statistically at par with each other (Table 3). From the two years of experiment it was observed that the tuber yield ranged from 16.50 to 21.58 and 16.82 to 21.86 t/ha during 2020–21 and 2021–22, respectively. Nevertheless, the tuber yield for rest of treatments varied from 17.27 to 19.03 and 17.57 to 19.29 t/ha during 2020–21 and 2021–22, respectively. The tuber yield enhancement of 30.8% was recorded during 2020–21 over control whereas, 29.9% tuber yield enhancement during 2021–22.

The relative potato tuber yield loss was influenced by the disease incidence and severity. During winter (*rabi*) season of 2020–21, the relative potato tuber yield loss was comparatively high because of congenial environmental conditions prevailed during the cropping season. It was observed that relative potato tuber yield loss ranged from 7.38 to 19.96% and 7.14 to 19.62% during 2020–21 and 2021–22, respectively (Table 3). T3 recorded least percent relative potato tuber yield loss of 7.38 and 7.16, whereas in control plot 23.54 and 23.04 during 2020–21 and 2021–22, respectively.

### 3.8 Economic analysis

Potato late blight disease slowly started reducing after the commencement of fungicides application thereby the tuber yield and BCR value increased. The highest BCR of 1:1.95 and 1: 1.99 with PLBDS of 8.81 and 5.96 were obtained from T7 followed by T3 (BCR:1:1.70,1: 1.75 and PLBDS: 14.63, 10.80) as compared to control (BCR: 1:1.48, 1: 1.53 and PLBDS: 14.63, 10.80) during 2020–21 and 2021–22, respectively (Table 4). The rest of the treatments ranged from 1.49 to 1.70 and 1.54 to 1.75 during 2020–21 and 2021–22, respectively. Additionally, T7 and T3 recorded 31.76%, 14.86% and 30.07%, 14.38% increase in BCR over control during 2020–21 and 2021–22, respectively. The highest gross income (Rs.3,88,380/-) and net income (Rs. 2,56,561/-) was found from T7 over control during 2020–21, whereas, gross income (Rs.3,93,480/-) and net income (Rs. 2,61,661/-) during 2021–22. Nonetheless, the net income enhancement of 30.84% and 29.94% was recorded with T7 over control during both the years, respectively.

**Table 4:** Economics of different treatments for the management of late blight in potato.

Treatments	Gross cost of cultivation (Rs/ha)				2020–21				2021–22			
					Gross Income** (Rs.)	Additional income/ha over control	Net Income (Rs.)	B:C ratio	Gross Income** (Rs.)	Additional income/ha over control	Net Income (Rs.)	B:C ratio
	Cost of cultivation	Treatments	Labour charges	Total (Rs.)								
T1	117500	5180	2000	125800	322140	25200	196340	1:1.56	328260	25440	202460	1:1.61
T2	117500	3902	2000	124680	310860	13920	186180	1:1.49	316260	13440	191580	1:1.54
T3	117500	7350	2000	133097	359700	62760	226603	1:1.70	365400	62580	232303	1:1.75
T4	117500	13597	2000	126850	316800	19860	189950	1:1.50	322140	19320	195290	1:1.54
T5	117500	12319	2000	135267	342600	45660	207333	1:1.53	347160	44340	211893	1:1.57
T6	117500	15767	2000	123402	334200	37260	210798	1:1.71	339300	36480	215898	1:1.75
T7	117500	6300	2000	131819	388380	91440	256561	1:1.95	393480	90660	261661	1:1.99
T8	117500	-	2000	119500	296940	-	177440	1:1.48	302820		183320	1:1.53

*Average of replications,

**-selling rates of potato @ Rs. 18000/t.

## 4. Discussion

Late blight disease is the most destructive disease of potato crop [11] and can spread through infected tuber [45,46], contaminated soil, wind [47]. The pathogen can spread rapidly from infected plant to healthy plant through air-borne sporangia, infected soil to uncontaminated soil through irrigation water and tools. At the time of harvesting, from the infected foliage the zoospores fall into soil and can survive in soil for many years [48]. Among the all management approaches, use of resistant cultivars are the most viable and eco-friendly option. However, due to the non availability of adequate quantity of seed materials, farmers are depends on susceptible cultivars with fungicides application [49]. Farmers are mostly relying on fungicides for the management because of high control efficacy, less risk of yield losses [49,50]. The fungicides used for the management of late blight disease are not completely reducing the disease [51] and the pathogen develops resistant against fungicides [52] and failure to reduce the disease [53]. Furthermore, it was evident that spraying of contact fungicide and contact + translaminar/systemic fungicide was more effective rather than application of single management option [33]. All the fungicides applied in experimental field were significantly suppressed the disease development as compared to control. Late blight disease development is influenced by different weather variables and positively correlated with maximum and minimum temperature. During the dry weather, the disease progress was slow whereas, after rain, because of evaporation the relative humidity increases in the environment which increases the disease progress. A maximum temperature ranges from 15.0–28.0°C and minimum temperatures ranges from 2.0–12.0°C were more suitable for late blight disease development in India [54]. Inference has been drawn by workers that the optimum temperature for development of late blight is 16.0–24.0°C, whereas sporangia are produced at temperature ranging between 5.6–26.0°C, with an optimum of 19.0–22.0°C [55], and when relative humidity is 90–100% [56]. Forecasting Models have been developed showing multiple linear regression (stepwise regression) which reveals that weather parameters would responsible for initiation and development of late blight of potato. Maximum temperature had significantly and positive correlated, whereas minimum relative humidity had significantly but negative correlation with disease severity of late blight [57]. AUDPC value was least in treated plot as compared to control plot. The AUDPC value increases with increasing crop age. Among the tested fungicides, mancozeb-cymoxanil + mancozeb, chlorothalonil-famoxadone + cymoxanil and chlorothalonil-ametoctradin + dimethomorph showed minimum late blight severity, AUDPC value with higher tuber yield and BCR value [49].

Potato late blight is polycyclic disease and spreads rapidly from infected plant to healthy plant. Moreover, prophylactic spray with Mandipropamid 23.4% SC (contact fungicide belongs to mandelamide class) followed by spraying with Ametoctradin 27% (contact fungicide belongs to pyrimidylamines class)+ Dimethomorph 20.27% SC (contact fungicide belongs to cinnamic acid amides class) effectively reduce the disease development. Foliar application of Ametoctradin 27% + Dimethomorph 20.27% SC @ 0.1% recorded terminal disease severity of 9.33 and 7.67%, yield 37.29 and 38.61 t/ha with disease control of 89.44 and 90.98% with no phytotoxic effect during 2009–10 and 2010–11, respectively [33]. Consequently, the application of contact fungicide before appearance of the disease followed by systemic + contact fungicide is effective approach to reduce the disease. This may be due to the inhibition of spore germination and to stop the penetration of germ tube. To prevent further infection, contact fungicide makes a layer on the leaves [58]. When the pathogen enters into epidermal cells through the formation of appressoria, then the contact fungicide became ineffective. Mandipropamid 23.4% SC inhibits the biosynthesis of phospholipids [59,60], growth of the pathogen [61] and most effective as a protectant fungicide against the disease [62,63]. If two different combination fungicides with different mode of action applied against *P*. *infestans*, it is very unlikely to build up resistant against both the fungicides at once [64]. In the present experiment, contact fungicide applied before the onset of the disease followed by systemic + contact fungicide. The findings of the present investigation are in agreement with the findings of several workers [32,50,65–68]. The prophylactic spray with contact fungicide followed by curative spray with systemic + contact fungicide effectively manages the late blight disease [33]. The mode of action of fungicide ametoctradin is inhibitor of respiratory in complex III, cytochrome bc1 of *P*. *infestans* [69,70]. It also affects the growth of *P*. *infestans*, zoospore production and release. The fungicide dimethomorph inhibit the sporulation [61], mycelia growth [71] and cell wall lysis in *P*. *infestans* [72–75]. The present experiment clearly indicated that foliar spraying of contact fungicide (35 & 55 DAS) followed by spraying with systemic + contact fungicide (45 & 65 DAS) gradually reducing the disease severity. The contact fungicide destroys the spores of the pathogen once came in contact and make a fungicide layer to protect the crop. Notably, a combination of systemic and contact fungicide will systematically enter into the plant cell and also directly protect the plant from the pathogen. The effective spray schedule with proper dose will reduce the number of sprays of fungicides of potato growers as well as cost of cultivation.

## 5. Conclusion

Late blight disease is an economically important disease of potato and causes qualitative as well as quantitative yield losses. In the present field experiment, the T7 found to be the most effective treatment against the hemibiotrophic pathogen, *P*. *infestans*, causing potato late blight disease which may be recommended to the farmer’s of the country. Foliar application of contact fungicide followed by systemic + contact fungicide have less chance to develop resistant against the serious disease under natural epiphytotic condition. Considering the impactness of the disease, further research can be taken up to develop late blight disease forecasting model to escape рrоbаble outbreaks and distribution of both the mating types (A1 and A2) through molecular-based technique to access genetic diversity in India.
